# Multiomics Dissection of Early‐Onset Colorectal Cancer (EOCRC) Reveals an Immunosuppressive Microenvironment and Identifies FAT3 Mutation as a Biomarker of Hot‐Like Immune Features

**DOI:** 10.1155/humu/8639334

**Published:** 2026-11-01

**Authors:** Liping Wang, Yanjun Lu, Weiyu Ma, Jinfang Guo, Wenbin Zhang, Yinan Shi

**Affiliations:** ^1^ Department of Colorectal Surgery Shanxi Province Cancer Hospital/Shanxi Hospital Affiliated to Cancer Hospital Chinese Academy of Medical Sciences/Cancer Hospital Affiliated to Shanxi Medical University Taiyuan China cacms.ac.cn; ^2^ Department of Gastroenterology Shanxi Province Cancer Hospital/Shanxi Hospital Affiliated to Cancer Hospital Chinese Academy of Medical Sciences/Cancer Hospital Affiliated to Shanxi Medical University Taiyuan China cacms.ac.cn; ^3^ Department of Endoscopy Shanxi Province Cancer Hospital/Shanxi Hospital Affiliated to Cancer Hospital Chinese Academy of Medical Sciences/Cancer Hospital Affiliated to Shanxi Medical University Taiyuan China cacms.ac.cn

**Keywords:** early-onset colorectal cancer (EOCRC), FAT3 mutation, immunosuppression, mutational signatures, tumor microenvironment (TME)

## Abstract

Early‐onset colorectal cancer (EOCRC) exhibits distinct clinical and biological features compared with late‐onset CRC (LOCRC), yet its genomic architecture and immune microenvironmental determinants remain incompletely characterized. Here, we integrated multiomics discovery analyses of 69 EOCRC and 461 LOCRC patients from the TCGA cohort, complemented by independent validation, single‐cell RNA sequencing, spatial transcriptomics, and systems‐level network analysis. We identified 10 genes significantly enriched for mutations in EOCRC, including ANXA7, ZNF267, and POLQ, which displayed distinct domain‐specific clustering and were validated in an external cohort. Mutational signature decomposition showed that EOCRC was associated with an SBS87‐like signature, whereas LOCRC was dominated by SBS1; because TCGA lacks detailed treatment history, this SBS87‐like inference should not be interpreted as direct evidence of prior thiopurine exposure. Immunologically, EOCRC displayed an immunosuppressive microenvironment characterized by elevated regulatory T cells, resting NK cells, and tighter immune‐stromal coupling, as further supported by spatial transcriptomic analysis. Single‐cell atlas analysis identified M2‐polarized, SPP1‐expressing macrophage subsets in EOCRC. Weighted gene coexpression network analysis identified a mitochondrial‐ribosomal biogenesis module as the transcriptional hallmark of EOCRC. Finally, random forest analysis identified FAT3 mutations as a genomic correlate of a hot‐like, immune‐activated phenotype in EOCRC. Integrative single‐cell analysis localized FAT3 predominantly to cancer‐associated fibroblasts, especially myofibroblasts, and an in silico FAT3 perturbation analysis suggested potential downstream programs involving extracellular matrix remodeling, inflammatory signaling, chemokine response, and mitotic spindle regulation. These findings delineate age‐stratified genomic and microenvironmental features of EOCRC and generate testable hypotheses for future functional and clinical validation.

## 1. Introduction

Colorectal cancer (CRC) remains a leading cause of cancer‐related morbidity and mortality worldwide [1]. Traditionally considered a disease of older adults, CRC has recently shown a troubling epidemiological shift: The incidence of early‐onset colorectal cancer (EOCRC), defined as diagnosis before the age of 50 years, has been rising steadily in many countries, whereas late‐onset colorectal cancer (LOCRC, diagnosed at age ≥ 50 years) is either stable or declining [2, 3]. This trend is particularly alarming because EOCRC often presents at advanced stages and exhibits distinct clinical and pathological features, including a higher likelihood of left‐sided or rectal primary tumors, signet‐ring histology, and a more aggressive clinical course even after adjusting for stage at diagnosis [4]. Despite extensive research, the molecular mechanisms that drive EOCRC remain incompletely understood, and most current treatment paradigms are extrapolated from older patients, potentially overlooking age‐specific biological vulnerabilities [5].

Emerging evidence suggests that EOCRC and LOCRC are not merely the same disease occurring at different ages but may arise through fundamentally different genetic and environmental pathways [6], whereas LOCRC is strongly associated with well‐established risk factors such as sedentary lifestyle, obesity, and dietary habits—and typically develops through the conventional adenoma–carcinoma sequence driven by APC, KRAS, TP53 mutations—EOCRC appears to have a distinct genomic landscape [7]. Several studies have reported that EOCRC tumors harbor fewer APC mutations but more frequent alterations in BRAF, CTNNB1, and DNA mismatch repair (MMR) genes, although findings are not entirely consistent [8]. Moreover, the contribution of hereditary syndromes (e.g., Lynch syndrome) accounts for only a fraction of EOCRC cases, indicating that sporadic early‐onset disease is driven by yet‐unknown somatic or germline factors [9, 10]. Beyond genetics, the tumor microenvironment (TME)—including immune cell infiltration, stromal activation, and metabolic reprogramming—may also differ with age of onset [11], but comprehensive comparisons between EOCRC and LOCRC at the multiomics level are still lacking.

Single‐cell and spatial transcriptomic technologies now offer unprecedented resolution to dissect intratumoral heterogeneity and cell–cell interactions [12–14]. Similarly, mutational signature analysis can deconvolute the etiological processes that have shaped a tumor′s genome, distinguishing endogenous (e.g., age‐related deamination) from exogenous (e.g., chemotherapy‐induced) mutagenesis [15, 16]. Applying these advanced analytical tools to age‐stratified CRC cohorts could reveal why younger patients develop cancer without the typical accumulation of age‐related mutations and what makes their tumors more aggressive.

In this study, we integrated bulk exome sequencing, bulk transcriptomics, single‐cell RNA‐sequencing (scRNA‐seq), and spatial transcriptomics from The Cancer Genome Atlas colorectal cancer (TCGA‐CRC) cohort, supplemented by an independent validation cohort (Dana–Farber Cancer Institute [DFCI]), the Single‐cell Colorectal Cancer Atlas, and the GSE144735 CRC single‐cell dataset. Our analyses were designed to address three interconnected objectives: (1) to delineate the differential mutational landscapes and mutational signatures between EOCRC and LOCRC, thereby uncovering age‐stratified carcinogenic processes; (2) to dissect the immune and stromal microenvironment at both bulk and single‐cell resolution, with particular emphasis on myeloid heterogeneity and immunosuppressive features; and (3) to identify coexpression networks that define the transcriptional hallmarks of EOCRC and to nominate genomic correlates—most notably FAT3 mutations—associated with immune hot‐like features in early‐onset CRC. By systematically comparing early‐onset and LOCRC across genomic, transcriptomic, and microenvironmental dimensions, we aim to establish a comprehensive molecular framework that illuminates potential mechanisms underlying EOCRC aggressiveness, prioritizes hypotheses for biomarker and therapeutic validation, and informs future age‐stratified precision‐oncology studies for the growing population of young CRC patients.

## 2. Materials and Methods

### 2.1. Data Sources

Bulk exome sequencing and transcriptomic data were obtained from TCGA‐CRC cohort [17], comprising 69 EOCRC patients (diagnosed at age < 50 years) and 461 LOCRC patients (diagnosed at age ≥ 50 years). Independent mutation validation was performed using the DFCI cohort [18]. scRNA‐seq data were retrieved from the Single‐cell Colorectal Cancer Atlas (https://crc.i-med.ac.at/). For the integrative FAT3 analysis, we additionally used the GSE144735 CRC scRNA‐seq dataset [19]. Spatial transcriptomics data were retrieved from publicly available CRC tissue sections. All data processing complied with relevant data usage policies and institutional guidelines.

### 2.2. scRNA‐seq Analysis

scRNA‐seq data were processed using the Seurat package (v4.0) [20]. Cells were filtered based on < 200 or > 7500 genes detected and > 20% mitochondrial reads. Log‐normalization was applied, followed by principal component analysis (PCA) dimensionality reduction. The Top 20 principal components were used for shared nearest neighbor (SNN) clustering (resolution = 0.5). Visualization was performed using uniform manifold approximation and projection (UMAP). Myeloid cells (macrophages, monocytes, and dendritic cells [DCs]) were subsetted using canonical lineage markers (CD14, FCGR1A, CD1C, CLEC10A, LILRA4, and CSF3R). M1 and M2 polarization scores were computed using signature genes (M1: CXCL9, CXCL10, CXCL11, IDO1, NOS2 and M2: CD163, MRC1, IL10, ARG1, SPP1). Violin plots were used to compare M1/M2 ratios across subclusters. Feature plots visualized expression of marker genes including CD163, MRC1, CD14, FCGR1A, CD1C, CLEC10A, LILRA4, JCHAIN, LAMP3, CSF3R, S100A8, S100A9, TOP2A, MKI67, and STMN1. Hierarchical clustering of subpopulation‐specific gene expression profiles was performed to identify functionally coherent myeloid groups.

### 2.3. Spatial Transcriptomics Analysis

Spatial transcriptomic data were generated using the 10x Visium platform. Alignment and gene expression quantification were performed with Space Ranger (v1.3). Spatial spots were aligned to histological images using the Seurat integration pipeline. Spatial distribution of T cell markers (CD3D, CD4, and CD8), fibroblast marker (COL1A1) and natural killer (NK) cell marker (GNLY) was visualized using SpatialFeaturePlot. Colocalization patterns of immune and stromal cells were compared between EOCRC and LOCRC tissue sections.

### 2.4. Weighted Gene Coexpression Network Analysis (WGCNA)

Bulk transcriptomic data from the TCGA‐CRC cohort were subjected to WGCNA using the WGCNA R package [21]. Genes with median absolute deviation in the Top 75% were retained. A soft‐thresholding power of *β* = 6 was selected based on scale‐free topology fit (*R* ^2^ ≥ 0.9). Coexpression modules were identified using dynamic tree cutting (minimum module size = 30, deepSplit = 3), and highly correlated modules (correlation > 0.8) were merged. Module eigengenes were calculated and correlated with EOCRC/LOCRC status. The lightgreen module, showing the highest positive correlation with EOCRC, was selected for downstream analysis. Gene ontology (GO) enrichment analysis was performed using clusterProfiler, covering biological processes, cellular components and molecular functions [22]. Enriched pathways included mitochondrial gene expression, ribosome biogenesis, lipid biosynthetic processes, protein localization to membrane, Notch receptor processing, methylation, post‐Golgi vesicle‐mediated transport, sterol biosynthesis, DNA‐templated transcription, glycoprotein biosynthesis, protein modification by small protein conjugation, heme biosynthesis, mitochondrion organization, positive regulation of telomere maintenance, ER‐nucleus signaling pathway, spliceosomal conformational changes, autophagy, nucleotide biosynthesis and regulation of cellular response to stress.

### 2.5. Functional Enrichment Analysis With Metascape

Functional enrichment analysis was performed using the Metascape platform (https://metascape.org) [23] to characterize the biological processes associated with the EOCRC‐specific gene module identified by WGCNA. Briefly, the list of genes was submitted to Metascape with *Homo sapiens* as the reference species. Enrichment analysis was restricted to Gene ontology biological process (GOBP) terms, as the primary interest was in pathway‐level functional interpretation. Statistical significance was assessed using a cumulative hypergeometric test (equivalent to Fisher′s exact test), and terms were considered significantly enriched if they met the criteria of *p* < 0.01, a minimum overlap of three genes between the query list and the annotated term, and an enrichment factor (observed/expected ratio) > 1.5. To reduce redundancy and facilitate biological interpretation, Metascape automatically clustered similar terms based on pairwise Kappa statistics; from each cluster, only the term with the smallest *p* value was retained as the representative. The resulting enriched GOBP terms were visualized as bar plots, with statistical significance expressed as –log_10_(*p*).

### 2.6. Mutation Data Analysis With Maftools

Somatic mutation MAF files were downloaded from TCGA. Analysis was performed using the maftools package (v2.10) [24]. Oncoplots were generated to visualize the top mutated genes by mutation type (missense, nonsense, splice site, frameshift insertions/deletions). Tumor mutational burden (TMB) was calculated as the total number of nonsilent mutations per megabase of coding sequence. The TMB panels in Figure 1C,D were used to provide pan‐cancer contextual visualization; no formal EOCRC‐versus‐LOCRC significance test is reported from these panels. Odds ratios (ORs) for mutation enrichment in EOCRC versus LOCRC were calculated using Fisher′s exact test; genes with p < 0.05 were considered significantly enriched. Co‐occurrence and mutual exclusivity of somatic mutations were assessed using the “somaticInteractions” function (*p* < 0.05). Lollipop plots for selected genes were generated to map the spatial distribution of mutations onto protein domains. Potentially druggable targets were annotated using OncoKB and drug–gene interaction databases.

Figure 1Genomic divergence between early‐onset and late‐onset colorectal cancer. (A) Oncoprint of the 20 most frequently mutated genes in EOCRC patients (*n* = 69), showing distinct mutation patterns compared with LOCRC. (B) Oncoprint of the 20 most frequently mutated genes in LOCRC patients (*n* = 461). (C,D) Pan‐cancer contextual distributions of tumor mutational burden (TMB) in EOCRC and LOCRC. These panels are descriptive and provide pan‐cancer context; no formal EOCRC‐versus‐LOCRC statistical comparison is presented. (E) Forest plot displaying odds ratios and 95% confidence intervals for 10 genes significantly enriched in EOCRC (*p* < 0.05, Fisher′s exact test). (F) Co‐occurrence matrix of somatic mutations in EOCRC, showing only two significant gene pairs (*p* < 0.05). (G) Co‐occurrence matrix in LOCRC, revealing 15 significant pairs, indicating more complex epistatic interactions. (H) List of potentially druggable targets annotated by OncoKB in the EOCRC cohort. (I) List of potentially druggable targets in the LOCRC cohort, revealing differential therapeutic vulnerabilities between age groups. *p < 0.01; ***p < 0.001.

**Figure:**
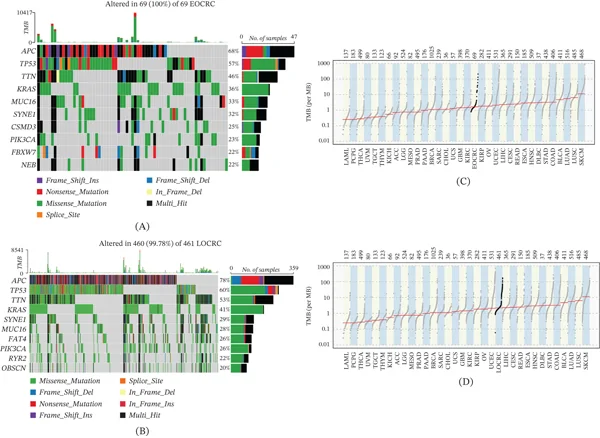

**Figure:**
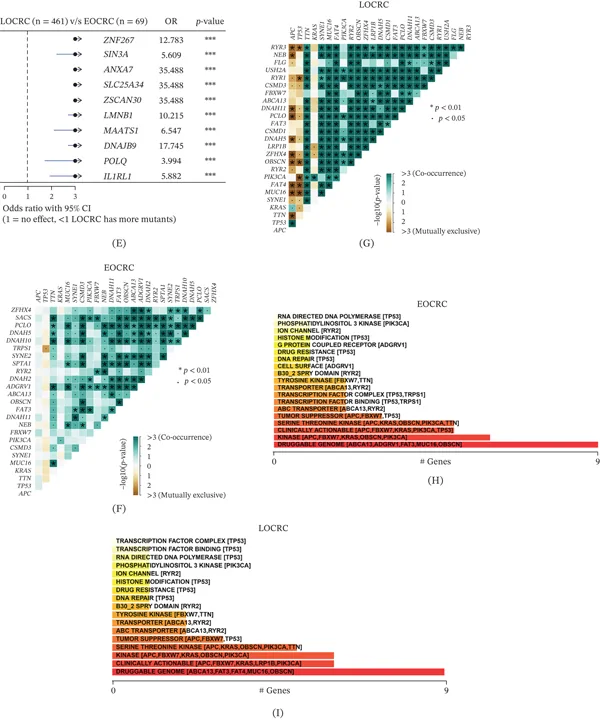

### 2.7. Mutational Signature Analysis

Single base substitution (SBS) contexts were extracted from TCGA MAF files to construct a 96‐trinucleotide mutation matrix. Nonnegative matrix factorization (NMF) was performed using the “sigminer” package (v2.0) [25]. The optimal number of signatures was determined by evaluating cophenetic correlation, dispersion and silhouette width. Extracted signatures were compared against COSMIC v3 reference signatures using cosine similarity [26]. Relative contributions of each signature were compared between EOCRC and LOCRC.

### 2.8. Immune Cell Infiltration Quantification

Bulk transcriptomic data (FPKM normalized) from the TCGA‐CRC cohort were used to estimate immune cell fractions with CIBERSORT (v1.03) [27]. The LM22 signature matrix (547 genes distinguishing 22 immune cell subtypes) was used with 1000 permutations. Only samples with CIBERSORT *p* < 0.05 were retained. Fractions of CD8+ T cells, regulatory T cells (Tregs) and resting NK cells were compared between EOCRC and LOCRC using the Mann–Whitney *U* test. Results with borderline *p* values were interpreted cautiously and were not treated as evidence of a clear absence of group differences. Immune scores and stromal scores were also computed using the xCELL algorithm [28], and Spearman′s correlation between the two scores was calculated separately for each group. Correlation coefficients were compared with assess differences in immune‐stromal coupling.

### 2.9. Hierarchical Clustering of Immune Phenotypes in the TCGA‐CRC Cohort

The enrichment levels of 29 immunogenomic features were quantified across 380 CRC samples from The Cancer Genome Atlas (TCGA‐CRC) cohort using single‐sample gene set enrichment analysis (ssGSEA) [29, 30]. The 29 immunogenomic features used for ssGSEA included adaptive immunity markers (CD8, CD4, Treg, Th1, Th2, and Th17), innate immunity markers (NK, macrophages, DCs, neutrophils), cytokine/chemokine signatures, immune checkpoint molecules (PD‐1, PD‐L1, CTLA‐4, LAG‐3, and TIM‐3), and other immune‐relevant gene sets. Unsupervised hierarchical clustering was subsequently performed on the resulting ssGSEA score matrix using Euclidean distance as the dissimilarity metric and Ward′s minimum variance method as the linkage criterion. This clustering analysis stratified the TCGA‐CRC cohort into two distinct immune phenotypic clusters, designated as cold‐like (immune‐depleted) and hot‐like (immune‐infiltrated) tumors, based on the relative enrichment patterns of the immune signatures.

### 2.10. Random Forest–Based Ranking of Mutation Importance

For EOCRC samples, somatic mutation data were retrieved from the TCGA‐CRC cohort, and genes with nonsynonymous mutation frequencies exceeding 15% were selected as candidate features. A random forest classifier was constructed to model the classification of EOCRC samples into hot‐like and cold‐like immune phenotypes based on the binary mutational status (mutant versus wild‐type) of these candidate genes. The importance of each gene was assessed by the mean decrease in Gini index, which quantifies the contribution of each variable to the homogeneity of nodes and leaves within the random forest. The Top 10 genes with the highest importance scores were extracted and ranked accordingly. The mutational frequencies of these top‐ranked genes were then compared between hot‐like and cold‐like EOCRC groups using Fisher′s exact test, and two‐tailed *p* values were calculated to determine the statistical significance of differential mutation distributions.

### 2.11. In Silico FAT3 Perturbation Analysis in Single‐Cell Data

For the integrative exploratory FAT3 analysis, we additionally analyzed the GSE144735 CRC scRNA‐seq dataset (six patients; 27,414 cells) [19]. Author‐provided cell‐type and subtype annotations were used after standardized quality control, normalization, highly variable gene selection, PCA, nearest‐neighbor graph construction, clustering, and t‐SNE visualization. FAT3 localization was assessed across major cell populations and CAF subtypes. Because FAT3 was most frequently detected in myofibroblast CAFs, the virtual knockout analysis was restricted to this subtype (*n* = 1226 cells; 90 FAT3‐positive and 1136 FAT3‐negative cells). A scTenifoldKnk‐inspired principal‐component regulatory‐network approach was used to compare wild‐type and in silico FAT3‐knockout networks [31]. Network perturbation was quantified as the row‐wise difference between wild type and knockout regulatory matrices, and significance was assessed against permuted random‐gene knockout null distributions with Benjamini–Hochberg correction. Predicted perturbation effects were compared with observed FAT3‐positive versus FAT3‐negative myofibroblast differential expression as an internal consistency check. GOBP enrichment was performed using Enrichr [32], and hallmark enrichment used the MSigDB hallmark collection [33]. This analysis was designed to generate hypotheses rather than establish causality.

### 2.12. Statistical Analysis

All statistical analyses were performed using R software (Version 4.1). Continuous variables between two groups (EOCRC vs. LOCRC) were compared using the Mann–Whitney *U* test unless otherwise specified. Categorical comparisons of mutation frequencies were evaluated using Fisher′s exact test, with ORs and 95% confidence intervals reported. For differential mutation enrichment across multiple genes, statistical significance was defined as *p* < 0.05, and high‐confidence hits were further annotated at *p* < 0.01. Correlation analyses between immune scores and stromal scores were conducted using Pearson′s correlation coefficient. For co‐occurrence and mutual exclusivity analyses of somatic mutations, *p* values were computed using Fisher′s exact test based on a 2 × 2 contingency table of mutation events. For multigroup and multifeature comparisons, multiple hypothesis correction was applied using the Benjamini–Hochberg false discovery rate (FDR) method, with an FDR threshold of < 0.05 considered statistically significant for enrichment analyses and module‐trait correlations. For the in silico FAT3 perturbation analysis, permutation‐derived *p* values were adjusted using the Benjamini–Hochberg method, and *q* < 0.10 was used to define the broad changed‐gene set for pathway‐level interpretation. Module scores between FAT3‐positive and FAT3‐negative myofibroblasts were compared using the Mann–Whitney *U* test. All reported *p* values are two‐sided. Statistical significance thresholds are indicated in the figures as  ^∗^ *p* < 0.05,  ^∗∗^ *p* < 0.01, and  ^∗∗∗^ *p* < 0.001.

## 3. Results

### 3.1. Genomic Divergence Between EOCRC and LOCRC in Age‐Stratified Mutational Landscapes

As a discovery analysis, we systematically compared the mutational profiles of 69 EOCRC patients (diagnosed at age < 50 years) against 461 LOCRC patients (diagnosed at age ≥ 50 years) from the TCGA‐CRC cohort. Oncoprint visualization of the top mutated genes revealed distinct mutational patterns between the two groups (Figure 1A,B). Although both EOCRC and LOCRC shared commonly mutated driver genes typical of colorectal malignancies, the overall distribution and co‐occurrence patterns differed notably between age‐defined subgroups.

We next assessed TMB in the context of pan‐cancer rankings. EOCRC and LOCRC showed broadly overlapping TMB distributions, with LOCRC displaying a marginally higher median TMB (Figure 1C,D). Because these panels are intended to provide pan‐cancer context rather than a formal age‐group comparison, we refrain from making a statistical significance claim here. These findings suggest that the biological distinction between EOCRC and LOCRC is unlikely to be explained solely by quantitative differences in mutational load and may instead involve qualitative differences in mutational repertoire.

To identify genes with differential mutation frequencies between EOCRC and LOCRC, we performed OR analysis. A forest plot revealed 10 genes significantly enriched for mutations in EOCRC compared with LOCRC, ranked by effect size and statistical significance (Figure 1E). Notably, ANXA7, SLC25A34, and ZSCAN30 exhibited the highest OR (OR = 35.488), followed by DNAJB9 (OR = 17.745), ZNF267 (OR = 12.783), LMNB1 (OR = 10.215), MAATS1 (OR = 6.547), SIN3A (OR = 5.609), IL1RL1 (OR = 5.882), and POLQ (OR = 3.994). All these genes reached statistical significance (*p* < 0.05), with most showing high confidence (*p* < 0.01), indicating that they represent genuine EOCRC‐predisposed mutational events rather than stochastic age‐related accumulation.

Subsequently, we investigated the co‐occurrence and mutual exclusivity patterns of somatic mutations within each subgroup. Interestingly, EOCRC displayed markedly fewer co‐occurring and mutually exclusive gene pairs compared with LOCRC (Figure 1F,G), suggesting a less structured or less cooperative oncogenic network in early‐onset disease. This relative paucity of epistatic mutational interactions may reflect distinct selective pressures operating in the TME of younger patients. Finally, we cataloged potentially druggable targets in both cohorts, revealing differential therapeutic vulnerabilities between EOCRC and LOCRC that may inform age‐stratified precision oncology strategies (Figure 1H,I).

### 3.2. Independent Validation of Recurrent Mutational Hotspots and Domain‐Specific Clustering in EOCRC‐Enriched Genes

Given the identification of 10 EOCRC‐enriched genes, we conducted lollipop plot analysis to delineate the spatial distribution and functional consequences of mutations within the seven most significant genes: ZNF267, SIN3A, ANXA7, SLC25A34, ZSCAN30, LMNB1, and POLQ. Across all seven genes, missense mutations constituted the predominant variant class, followed by frameshift insertions/deletions (Figure 2A–G). These mutation patterns were distributed nonrandomly along the protein sequences, with several genes exhibiting clustering at specific functional domains, indicative of positive selection and potential driver roles in EOCRC pathogenesis.

**Figure 2:**
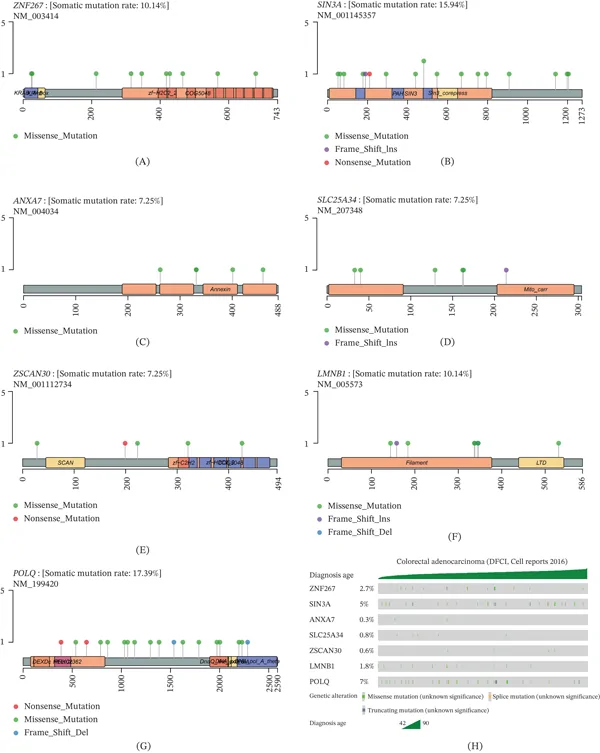
Recurrent mutational hotspots and domain‐specific clustering in EOCRC‐enriched genes. (A) Lollipop plot of *ZNF267* mutations, with missense variants clustering within the KRAB‐A and zinc‐finger domains. (B) Lollipop plot of *SIN3A* mutations, showing enrichment in the paired amphipathic helix (PAH) domains. (C) Lollipop plot of *ANXA7* mutations, with several frameshift deletions in the annexin repeat region. (D) Lollipop plot of *SLC25A34* mutations, predominantly missense changes distributed across mitochondrial carrier domains. (E) Lollipop plot of *ZSCAN30* mutations, with clustering in the SCAN box and zinc‐finger motifs. (F) Lollipop plot of *LMNB1* mutations, showing nonrandom distribution along the lamin tail domain. (G) Lollipop plot of *POLQ* mutations, with helicase and polymerase domains harboring multiple missense and truncating events. (H) Validation in the independent DFCI cohort confirms that mutation frequencies of these seven genes are significantly lower in older CRC patients.

To validate the age‐dependent enrichment of these mutations, we interrogated an independent external cohort from the DFCI (Cell Reports 2016). Comparative analysis confirmed that the mutation frequencies of ZNF267, SIN3A, ANXA7, SLC25A34, ZSCAN30, LMNB1, and POLQ were substantially lower in older CRC patients (Figure 2H), corroborating their specificity to the early‐onset context. The concordance between the TCGA discovery cohort and the DFCI validation cohort—confirming that the mutation frequencies of these genes were substantially lower in older CRC patients—supports the age‐specific relevance of these candidate EOCRC biomarkers. However, the DFCI cohort was not designed to replicate the full EOCRC versus LOCRC comparison using identical age cutoffs, and therefore this validation should be considered supportive rather than confirmatory.

### 3.3. An SBS87‐Like Signature in EOCRC Versus Clock‐Like Cytosine Deamination in LOCRC

To elucidate the underlying mutational processes operative in EOCRC versus LOCRC, we deconvoluted the SBS profiles using NMF. The 96‐class substitution profiles revealed distinct compositional patterns between the two age groups (Figure 3A,D). For EOCRC, NMF decomposition metrics—including cophenetic correlation, dispersion, and silhouette analysis—collectively indicated that four mutational signatures optimally explained the observed mutational spectrum (Figure 3B). The most prominent signature in EOCRC showed similarity to SBS87 (cosine similarity = 0.732; Figure 3C). Because TCGA lacks detailed records of prior thiopurine exposure, inflammatory bowel disease history, and treatment timing, we refer to this signal as an SBS87‐like signature and do not interpret it as direct evidence of prior chemotherapy exposure.

Figure 3Mutational signature analysis reveals an SBS87‐like pattern in EOCRC versus clock‐like deamination in LOCRC. (A) Ninety‐six‐trinucleotide substitution profiles of EOCRC tumors, showing a relatively flat distribution with slight C > T enrichment. (B) NMF decomposition metrics (cophenetic correlation, dispersion, silhouette width) for EOCRC, collectively supporting a four‐signature solution. (C) Relative contribution of each mutational signature in EOCRC, with an SBS87‐like signature being the most dominant (cosine similarity = 0.732); because treatment history is unavailable, this is interpreted as an SBS87‐like pattern rather than direct evidence of thiopurine exposure. (D) Ninety‐six‐trinucleotide substitution profiles of LOCRC tumors, dominated by C > T transitions at CpG dinucleotides. (E) NMF decomposition metrics for LOCRC, also selecting four signatures as optimal. (F) Relative contribution of each signature in LOCRC, where SBS1 (spontaneous 5‐methylcytosine deamination) is the most dominant (cosine similarity = 0.957).

**Figure:**
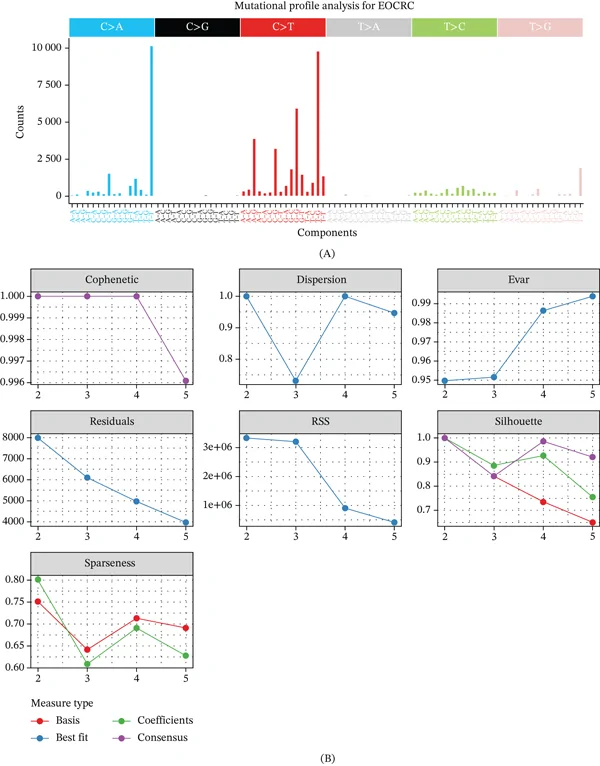

**Figure:**
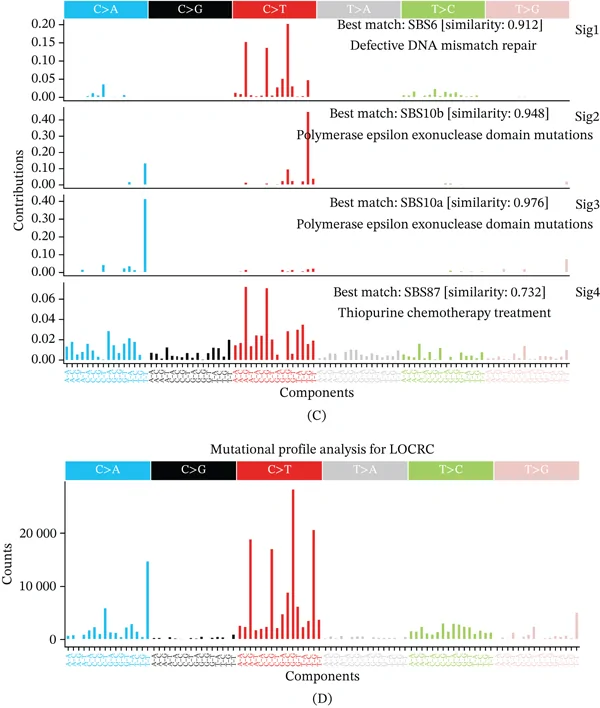

**Figure:**
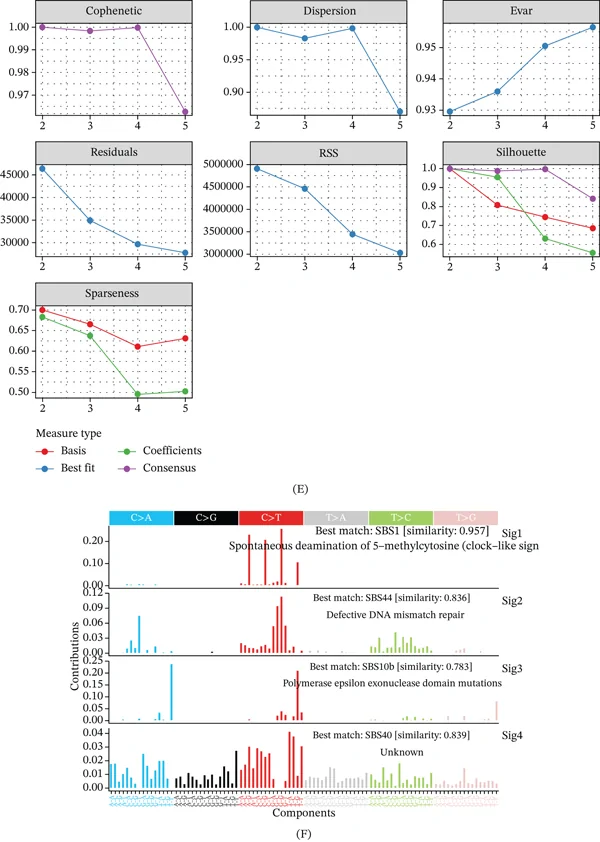

In contrast, LOCRC also exhibited an optimal signature number of four (Figure 3E), but was dominated by SBS1 (cosine similarity = 0.957), corresponding to spontaneous deamination of 5‐methylcytosine (Figure 3F). This signature is a well‐established hallmark of age‐related cumulative mutational damage, consistent with the older age distribution of the LOCRC cohort. Additional signatures identified in both groups included those associated with defective DNA MMR (SBS6, SBS44) and polymerase epsilon exonuclease domain mutations (SBS10a, SBS10b), though their relative contributions varied between EOCRC and LOCRC. These data indicate that EOCRC and LOCRC are associated with different mutational‐signature patterns—an SBS87‐like signal in EOCRC versus age‐dependent deamination in LOCRC—providing exploratory clues to their divergent etiologies.

### 3.4. An Immunosuppressive TME Characterized by Treg Enrichment, Resting NK Cells, and Tight Immune‐Stromal Coupling in EOCRC

Transcending the genomic level, we next investigated whether EOCRC exhibits distinct immune microenvironment features. Using bulk tissue transcriptomic data deconvoluted by CIBERSORT, CD8+ T‐cell fractions showed a borderline difference between EOCRC and LOCRC (*p* = 0.0497; Figure 4A), which we therefore interpret cautiously rather than as evidence of a clear absence of difference. Treg scores were significantly elevated in EOCRC (*p* = 3.58*e* − 02; Figure 4B), alongside a significant enrichment of resting NK cells (*p* = 1.46*e* − 02; Figure 4C). Together, these findings suggest an immunosuppressive skew in EOCRC, characterized by enhanced Treg‐mediated tolerance and altered NK cell activation states, whereas the modest and borderline CD8+ T‐cell difference should not be overinterpreted.

**Figure 4:**
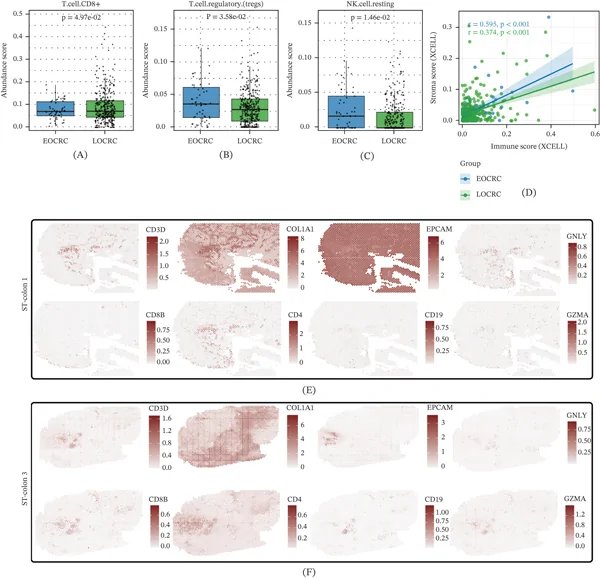
Immunosuppressive tumor microenvironment in EOCRC characterized by Treg enrichment, resting NK cells, and tight immune–stromal coupling. (A) CD8+ T‐cell fractions showed a borderline difference between EOCRC and LOCRC (*p* = 0.0497). (B) Regulatory T‐cell (Treg) scores are significantly elevated in EOCRC compared with LOCRC (*p* = 0.0358). (C) Resting natural killer (NK) cell fractions are significantly higher in EOCRC than in LOCRC (*p* = 0.0146). (D) Immune–stromal correlation (xCELL) is significantly stronger in EOCRC (*r* = 0.595, *p* < 0.001) than in LOCRC (*r* = 0.374, *p* < 0.001). (E) Spatial distribution of EPCAM, CD3D, CD4, CD8/CD8B, CD19, COL1A1, GNLY, and GZMA in EOCRC tissue sections, including the fibroblast marker COL1A1. (F) Spatial distribution of the same marker panel in LOCRC tissue sections, allowing comparison of T‐cell markers, COL1A1‐expressing fibroblasts, and NK/cytotoxicity markers between groups.

Furthermore, correlation analysis between immune scores and stromal scores (assessed by xCELL) revealed a significantly stronger positive correlation in EOCRC compared with LOCRC (EOCRC: *r* = 0.595, *p* < 0.001; LOCRC: *r* = 0.374, *p* < 0.001; Figure 4D), indicating tighter coupling between immune infiltration and desmoplastic stromal reaction in early‐onset disease.

To spatially resolve these observations, we performed spatial transcriptomic analysis. Visualization of epithelial, T‐cell, B‐cell, fibroblast, and NK/cytotoxicity markers (EPCAM, CD3D, CD4, CD8/CD8B, CD19, COL1A1, GNLY, and GZMA) revealed distinct spatial architectures between EOCRC and LOCRC tissue sections (Figure 4E,F). EOCRC tissues exhibited altered spatial distribution patterns with notable enrichment of COL1A1‐expressing fibroblasts and differential clustering of immune cell subsets, suggesting that the EOCRC microenvironment is characterized by both quantitative immune suppression and qualitative spatial reorganization of stromal‐immune interactions.

### 3.5. Myeloid Heterogeneity Reveals M2‐Polarized, SPP1‐Expressing Macrophage Subsets in EOCRC

Given the observed immunological differences, we dissected the myeloid compartment at single‐cell resolution. UMAP clustering of macrophages, monocytes, and DCs revealed multiple transcriptionally distinct subpopulations (Figure 5A), each characterized by unique marker gene expression profiles (Figure 5B). We then computed M1 (proinflammatory) and M2 (anti‐inflammatory/tissue repair) polarization signatures across the myeloid landscape. Global M1 and M2 scores showed variable distribution across the UMAP embedding (Figure 5C), with distinct subpopulations exhibiting differential polarization states.

Figure 5Single‐cell atlas of myeloid heterogeneity reveals M2‐polarized, SPP1‐expressing macrophage subsets in EOCRC. (A) UMAP embedding of myeloid cells (macrophages, monocytes, and dendritic cells) from EOCRC samples, revealing eight transcriptionally distinct subpopulations. (B) Heatmap of marker gene expression across each subpopulation, confirming identities such as CD14+ classical monocytes, CD1C+ dendritic cells, and CD163+ macrophages. (C) Global M1 (proinflammatory) and M2 (anti‐inflammatory) signature scores projected onto the UMAP, showing variable polarization across clusters. (D) Violin plots comparing M1/M2 polarization ratios across subclusters, with two macrophage clusters exhibiting significantly higher M2 dominance. (E) Feature plots of canonical lineage and proliferation markers (CD163, MRC1, CD14, FCGR1A, TOP2A, MKI67) validating the identity and proliferative status of subpopulations. (F) Hierarchical clustering of subpopulation‐specific gene expression profiles, grouping myeloid cells into functionally coherent categories including inflammatory monocytes, regulatory DCs, and M2‐like macrophages. (G) Reciprocal expression patterns of the M1‐associated chemokine CXCL9 and the M2‐associated osteopontin SPP1 across the myeloid landscape, with SPP1 highly enriched in two macrophage clusters.

**Figure:**
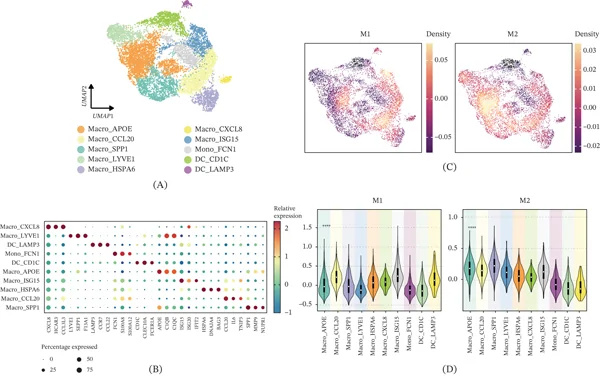

**Figure:**
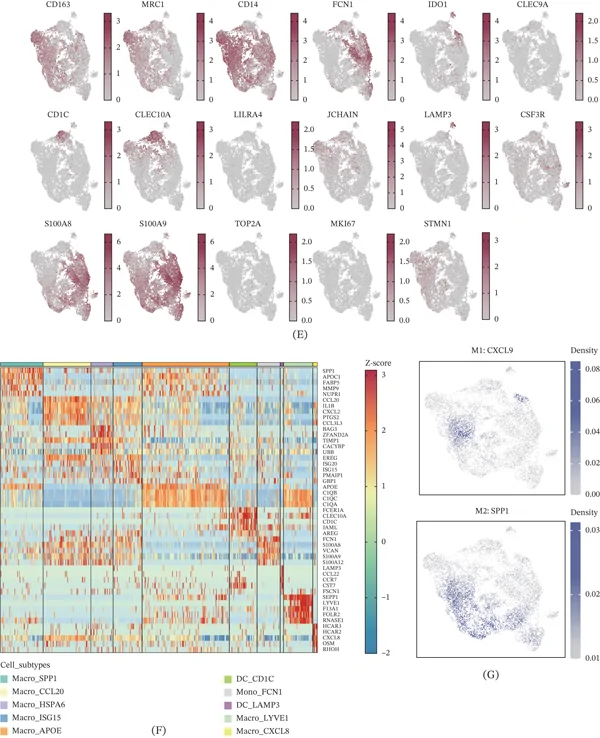

Violin plot decomposition demonstrated that specific macrophage and monocyte subclusters harbored divergent M1/M2 polarization ratios (Figure 5D), suggesting substantial functional heterogeneity within the EOCRC myeloid compartment. Feature plots of canonical lineage markers—including CD163, MRC1, CD14, FCGR1A, CD1C, CLEC10A, LILRA4, JCHAIN, LAMP3, CSF3R, S100A8, S100A9, TOP2A, MKI67, and STMN1—confirmed the identity and proliferative status of these subpopulations (Figure 5E). Hierarchical clustering of subpopulation‐specific gene expression further stratified myeloid cells into functionally coherent subgroups (Figure 5F).

Notably, examination of established M1 and M2 discriminatory genes revealed that CXCL9 (M1‐associated) and SPP1 (M2‐associated) exhibited reciprocal expression patterns across the myeloid landscape (Figure 5G). The presence of SPP1‐expressing myeloid subsets in EOCRC is consistent with the protumorigenic, immunosuppressive microenvironment inferred from our bulk transcriptomic analyses and suggests that myeloid‐derived suppression may contribute to EOCRC progression.

### 3.6. Integrative Systems‐Level Analysis Identifies a Mitochondrial‐Ribosomal Biogenesis Module as the Transcriptional Hallmark of EOCRC

To identify systems‐level transcriptional programs distinguishing EOCRC from LOCRC, we performed WGCNA analysis. Scale‐free topology analysis established a soft‐thresholding power of *β* = 6 as the optimal parameter for network construction (Figure 6A). This analysis partitioned the transcriptome into 29 distinct coexpression modules (Figure 6B), each represented by a unique color identifier.

Figure 6Systems‐level network analysis identifies a mitochondrial‐ribosomal biogenesis module as the transcriptional hallmark of EOCRC. (A) Scale‐free topology fit for soft‐thresholding powers, with *β = 6* selected as the optimal value achieving an *R* ^2^ > 0.9. (B) Dendrogram of coexpression modules identified by WGCNA, with 29 distinct modules color‐coded and the lightgreen module highlighted. (C) Average gene significance across all modules for EOCRC versus LOCRC status, showing that the lightgreen module has the highest positive significance. (D) Hierarchical clustering of module eigengenes and correlation heatmap, demonstrating that the lightgreen module strongly correlates with EOCRC. (E) Module–trait correlation plot confirming the lightgreen module as the most positively associated with EOCRC, whereas several other modules correlate with LOCRC. (F) Gene Ontology enrichment analysis of the lightgreen module reveals significant overrepresentation of mitochondrial gene expression, ribosome biogenesis, lipid biosynthetic process, sterol biosynthesis, and protein localization to membrane, among others. (G) Term nodes are colored by cluster identity. (H) Same network colored by –log_10_(*p*) (darker orange means more significant).

**Figure:**
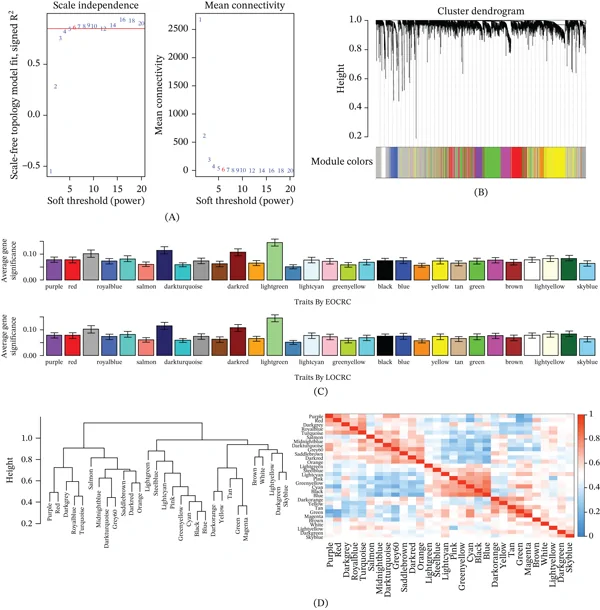

**Figure:**
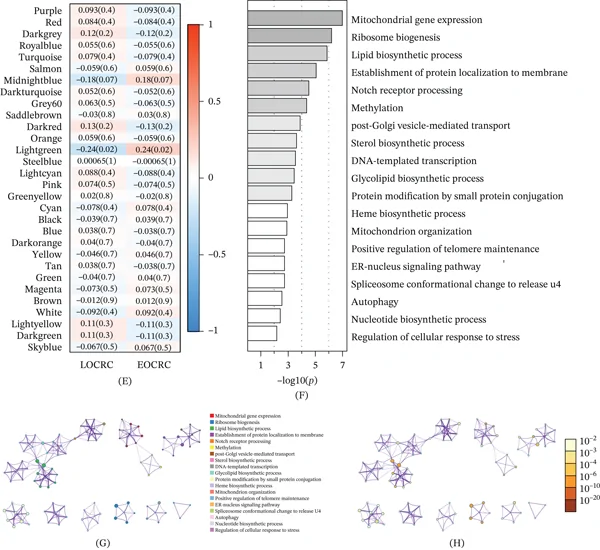

We quantified the module‐trait relationships by calculating average gene significance for EOCRC versus LOCRC status across all 29 modules (Figure 6C). Hierarchical clustering of module eigengenes and their corresponding correlation heatmap revealed that several modules exhibited strong differential association with age of onset (Figure 6D). Strikingly, the lightgreen module demonstrated the highest positive correlation with the EOCRC trait (Figure 6E), identifying it as the most EOCRC‐specific transcriptional program.

GO and pathway enrichment analysis of the lightgreen module revealed significant enrichment in mitochondrial gene expression, ribosome biogenesis, lipid biosynthetic processes, establishment of protein localization to membrane, Notch receptor processing, methylation, post‐Golgi vesicle‐mediated transport, sterol biosynthetic process, DNA‐templated transcription, glycoprotein biosynthetic process, protein modification by small protein conjugation, heme biosynthetic process, mitochondrion organization, positive regulation of telomere maintenance, ER‐nucleus signaling pathway, spliceosomal conformational changes, autophagy, nucleotide biosynthetic process, and regulation of cellular response to stress (Figure 6F). To visualize the GOBP enrichment results of the EOCRC‐specific lightgreen module, we constructed term–term similarity networks. When colored by cluster identity, the network revealed several functionally coherent groups, with the largest cluster centered on mitochondrial gene expression and ribosome biogenesis, followed by clusters related to lipid/sterol biosynthesis, protein localization, and telomere maintenance (Figure 6G). Coloring the same network by –log_10_(*p*) showed that mitochondrial and ribosomal biogenesis terms exhibited the highest significance, acting as central hubs (Figure 6H). The enrichment of mitochondrial and ribosomal biogenesis pathways, coupled with lipid and sterol biosynthesis, suggests that EOCRC is metabolically reprogrammed toward anabolic processes and oxidative phosphorylation. These network‐level findings provide a mechanistic framework linking the distinct mutational and immune profiles of EOCRC to underlying metabolic vulnerabilities that may represent novel therapeutic targets for this aggressive, early‐onset malignancy.

### 3.7. FAT3 Mutations as a Candidate Genomic Biomarker of the Hot‐Like Immune Phenotype in EOCRC

To elucidate the genomic determinants associated with immune phenotypic heterogeneity in EOCRC, we first performed unsupervised hierarchical clustering of 380 CRC samples from the TCGA cohort based on ssGSEA scores of 29 immunogenomic features encompassing adaptive immunity, innate immunity, cytokine signaling, and immune checkpoint molecules. This discovery analysis classified tumors into two distinct immune archetypes: cold‐like and hot‐like (Figure 7A). The cold‐like and hot‐like clusters exhibited different enrichment patterns across all 29 immune features. Specifically, cold‐like tumors showed lower expression of CD8+ T‐cell markers, IFN‐*γ* signaling, and immune checkpoint molecules, whereas hot‐like tumors demonstrated coordinated upregulation of these immune‐activating features. Stacked bar chart analysis subsequently demonstrated that the hot group contained a substantially higher proportion of LOCRC compared with the cold group, suggesting an age‐dependent divergence in immune landscape patterning (Figure 7B).

Figure 7FAT3 mutations are associated with an immune hot‐like phenotype in early‐onset colorectal cancer. (A) Unsupervised hierarchical clustering of ssGSEA scores for 29 immunogenomic features across 380 TCGA‐CRC samples stratified the cohort into two distinct immune phenotypes, designated cold‐like and hot‐like. (B) Stacked bar chart analysis revealed that the hot‐like group exhibited a substantially higher proportion of late‐onset colorectal cancer (LOCRC) compared with the cold‐like group. (C) Random forest algorithm‐based ranking of genes with mutation frequencies exceeding 15% in EOCRC identified the Top 10 most important genes for distinguishing hot‐like from cold‐like tumors, with APC, FAT3, and TRPS1 exhibiting the highest mean decrease in Gini importance scores. (D) Bar plots comparing the mutational status of the Top 10 ranked genes between hot‐like and cold‐like EOCRC demonstrated significantly differential distributions for all genes except TP53. (E) The forest plot illustrates the odds ratios (ORs) of mutated genes comparing cold‐like and hot‐like EOCRC; FAT3 exhibited the highest OR, ranking first. (F) Lollipop plots illustrating the distribution of FAT3 mutations showed that missense and frameshift alterations were predominantly enriched in hot‐like EOCRC (40.74%; *n* = 27) compared with cold‐like EOCRC (3.57%; *n* = 28). (G) Box plot comparisons between FAT3‐mutated and wild‐type EOCRC revealed significant differences in mutation–immune‐related features, including SNV neoantigens, nonsilent mutation rate, indel neoantigens, fraction altered, aneuploidy score, number of segments, homologous recombination defects, leukocyte fraction, IFN‐*γ* response, and macrophage regulation. **p < 0.01; ***p < 0.001.

**Figure:**
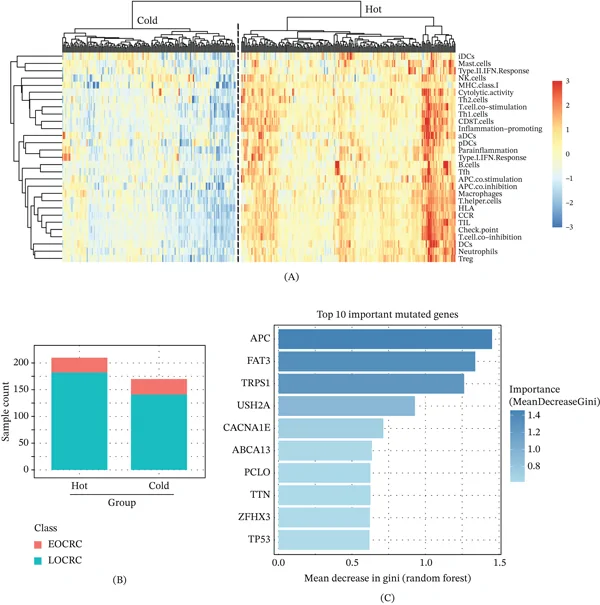

**Figure:**
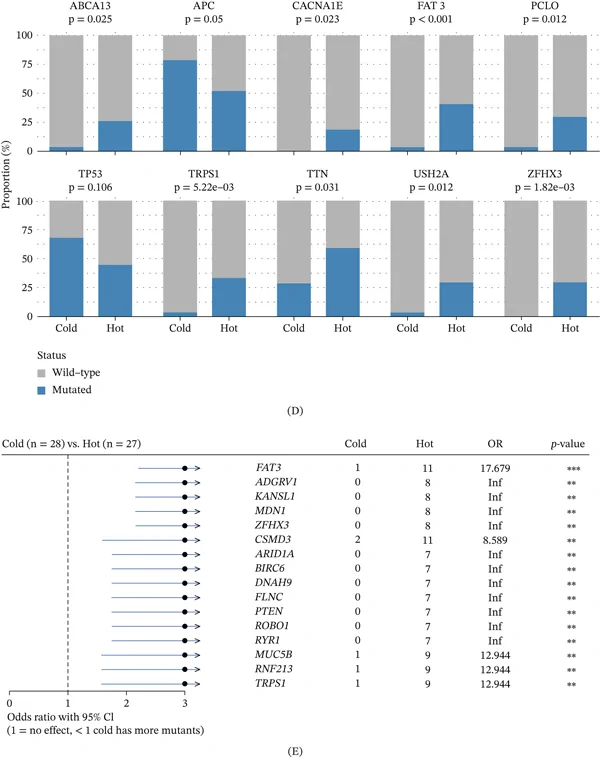

**Figure:**
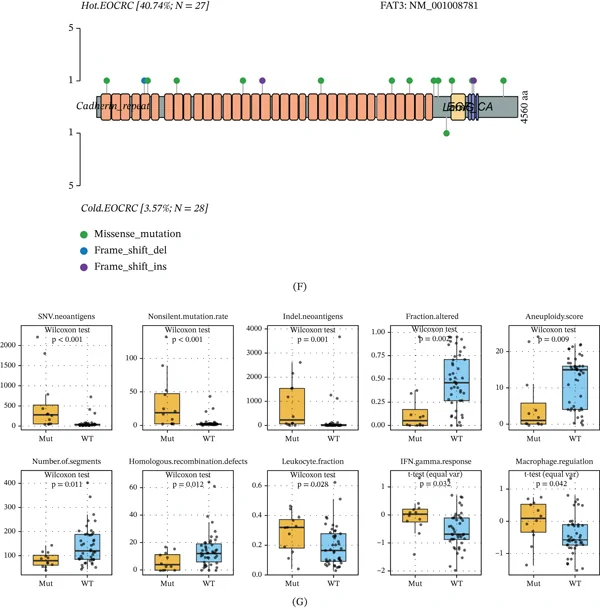

To identify somatic mutations associated with this immune dichotomy specifically in EOCRC, we applied a random forest algorithm to rank the discriminative importance of genes with mutation frequencies exceeding 15%. Among the Top 10 most important genes, APC, FAT3, and TRPS1 exhibited the highest mean decrease in Gini importance scores (Figure 7C). Analysis of the mutational status across these Top 10 genes revealed that, except for TP53, all other genes—including ABCA13, CACNA1E, PCLO, USH2A, TTN, and ZFHX3—showed significantly differential distributions between hot‐ and cold‐like EOCRC, with hot‐like tumors generally harboring higher mutation frequencies (Figure 7D). Although APC ranked highest in importance, its well‐established role as a ubiquitous CRC driver across all ages made it a less compelling candidate for the EOCRC‐specific immune‐phenotype association. Conversely, FAT3—which ranked second—has not been previously characterized as a major driver in EOCRC, and its enrichment in hot‐like EOCRC (40.74% vs. 3.57%) suggested a potentially EOCRC‐specific immune‐modulating association. We therefore prioritized FAT3 for further investigation as a hypothesis‐generating observation. The forest plot displays the ORs of mutated genes between cold‐like EOCRC and hot‐like EOCRC, showing only genes with *p* values < 0.01; among them, FAT3 mutation has the highest OR in hot‐like tumors (Figure 7E). Lollipop plot visualization indicated that FAT3 alterations were predominantly missense mutations and frameshift deletions/insertions and were enriched in hot‐like EOCRC (40.74%; *n* = 27) relative to cold‐like EOCRC (3.57%; *n* = 28) (Figure 7F). Comparative analysis between FAT3‐mutated and wild‐type EOCRC revealed that FAT3 mutations were associated with elevated SNV neoantigens, nonsilent mutation rate, indel neoantigens, fraction altered, aneuploidy score, number of segments, and homologous recombination defects, alongside enhanced leukocyte fraction, IFN‐*γ* response, and macrophage regulation (Figure 7G). Among these associations, elevated neoantigen burden and homologous recombination defects provide a plausible—but not yet experimentally demonstrated—link between FAT3‐mutant tumors and hot‐like immune features. This association may reflect broader genomic instability rather than a direct causal effect of FAT3 loss. Collectively, these findings nominate FAT3 mutation as a candidate genomic correlate of a hot‐like, immune‐activated phenotype in EOCRC and provide a testable hypothesis for future functional studies.

### 3.8. Integrative Single‐Cell Analysis Localizes FAT3 to Myofibroblast CAFs and Supports In Silico Perturbation

To further contextualize the potential cellular compartment and downstream programs associated with FAT3, we analyzed the GSE144735 CRC scRNA‐seq dataset [19]. After standardized preprocessing and author‐guided annotation, 27,414 cells from six patients were embedded by t‐SNE (Figure 8A), spanning normal, border, and tumor tissues (Figure 8B), and six major compartments: CAF (*n* = 7650; 27.91%), epithelial cells (*n* = 6168; 22.50%), T cells (n = 5,770; 21.05%), B cells (n = 4,902; 17.88%), myeloid cells (*n* = 2676; 9.76%), and mast cells (*n* = 248; 0.90%) (Figure 8C,D). FAT3 was rarely detected in epithelial cells and was concentrated predominantly in the CAF region (Figure 8E). Among CAF subtypes, myofibroblasts showed the highest FAT3‐positive fraction (90/1226; 7.34%) (Figure 8F), providing a rationale for modeling perturbation in this compartment. FAT3‐positive myofibroblasts exhibited higher ECM/collagen (*p* = 2.1*e* − 14), MMP/remodeling (*p* = 2.2*e* − 06), EMT (*p* = 2.5*e* − 10), and angiogenesis (*p* = 2.9*e* − 11) module scores than FAT3‐negative myofibroblasts (Figure 8G). Using a scTenifoldKnk‐inspired regulatory‐network perturbation approach [31], in silico FAT3 knockout in myofibroblasts yielded 182 significantly changed genes (Benjamini–Hochberg *q* < 0.10). Among the Top 30 perturbation‐ranked genes, 89% showed predicted knockout effects opposite to the observed FAT3‐positive state (Figure 8H), indicating internal consistency between the perturbation model and observed expression patterns but not establishing causality. GOBP enrichment of all changed genes highlighted chemokine‐mediated signaling, cellular response to chemokines, neutrophil chemotaxis and migration, and spindle‐related processes (Figure 8I). MSigDB hallmark analysis additionally highlighted TNF‐*α* signaling via NF‐*κ*B, inflammatory response, interferon gamma response, complement, E2F targets, and mitotic spindle programs (Figure 8J). These integrative analyses are exploratory and hypothesis generating, and experimental perturbation in CAF or tumor‐CAF coculture systems will be required to test the predicted effects.

Figure 8Integrative single‐cell analysis and in silico FAT3 perturbation in myofibroblast CAFs. (A) t‐SNE embedding of 27,414 cells from six CRC patients in GSE144735, colored by patient. (B) t‐SNE embedding colored by tissue source (normal, border, and tumor). (C) Six major cell groups: CAF (*n* = 7650), epithelial cells (*n* = 6168), T cells (*n* = 5770), B cells (*n* = 4902), myeloid cells (*n* = 2676), and mast cells (*n* = 248). (D) Dot plot of canonical marker genes supporting major cell‐group annotation. (E) FAT3‐expressing cells are concentrated predominantly in the CAF region of the t‐SNE embedding. (F) FAT3‐positive fractions across CAF subtypes; myofibroblasts show the highest FAT3‐positive fraction (90/1226; 7.34%). (G) Module‐score comparison between FAT3‐positive and FAT3‐negative myofibroblasts: ECM/collagen (*p* = 2.1*e* − 14), MMP/remodeling (*p* = 2.2*e* − 06), EMT (*p* = 2.5*e* − 10), and angiogenesis (*p* = 2.9*e* − 11). (H) Top 30 perturbation‐ranked genes after in silico FAT3 knockout; 89% show predicted knockout effects opposite to the observed FAT3‐positive state. The broader predicted‐versus‐observed effect concordance was Spearman rho = 0.498 (*p* = 2*e* − 87). (I) Gene ontology biological process enrichment of all significantly changed genes after virtual FAT3 knockout (*n* = 182), highlighting chemokine signaling, neutrophil chemotaxis/migration, and spindle‐related processes. (J) MSigDB hallmark enrichment of the same changed‐gene set, highlighting TNF‐*α* signaling via NF‐*κ*B, inflammatory response, interferon gamma response, complement, E2F targets, and mitotic spindle programs.

**Figure:**
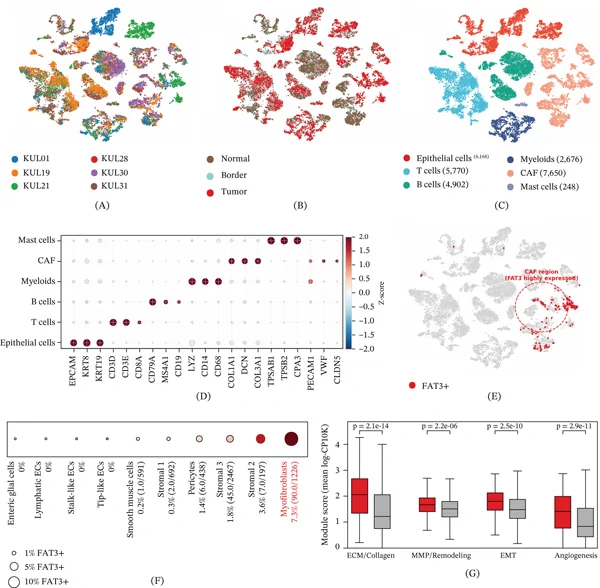

**Figure:**
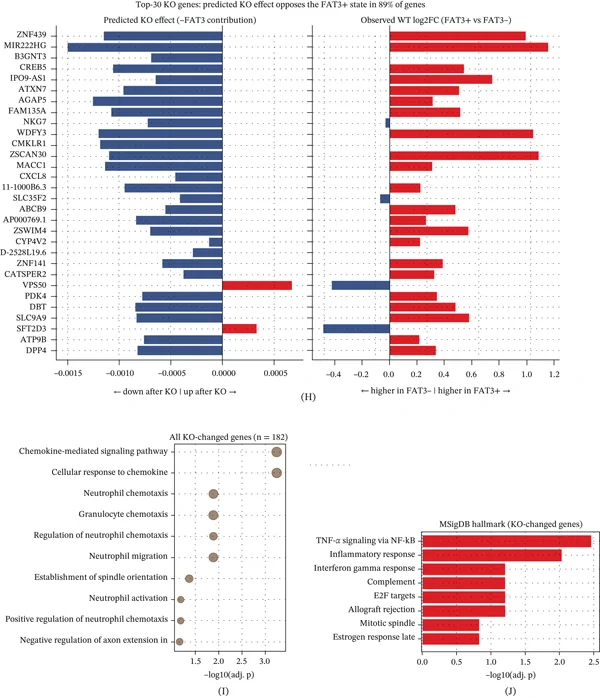

## 4. Discussion

In this multiomics study, we systematically compared the genomic landscapes, mutational processes, immune microenvironment, and transcriptional programs of EOCRC and LOCRC. To make the evidence hierarchy explicit, TCGA‐based genomic and immune analyses constitute discovery analyses; the DFCI cohort provides independent validation; spatial transcriptomics, single‐cell analyses, and WGCNA provide integrative or exploratory support; and the FAT3 association together with the in silico perturbation analysis is hypothesis generating. Our findings indicate that EOCRC is not simply an earlier manifestation of the same disease but rather a molecularly heterogeneous entity with distinct genomic and microenvironmental features.

At the genomic level, descriptive pan‐cancer TMB visualization showed broadly overlapping distributions between EOCRC and LOCRC; however, we identified several genes that are significantly mutated in EOCRC, including ANXA7, SLC25A34, ZSCAN30, DNAJB9, ZNF267, LMNB1, MAATS1, SIN3A, IL1RL1, and POLQ. Several of these—such as the transcriptional corepressor SIN3A, the DNA repair gene POLQ [34], and the zinc‐finger protein ZNF267—have not been previously highlighted as major drivers in EOCRC, suggesting novel mechanisms of early‐onset colon carcinogenesis. Lollipop plots revealed nonrandom clustering of missense mutations within functional domains, indicating positive selection. Moreover, EOCRC displayed markedly fewer co‐occurring and mutually exclusive mutation pairs than LOCRC, implying that early‐onset tumors may rely on a smaller set of potent genetic drivers rather than complex epistatic networks.

Mutational signature analysis provides an exploratory explanation for this age‐dependent divergence. LOCRC was dominated by SBS1, the clock‐like signature resulting from spontaneous deamination of 5‐methylcytosine, which accumulates linearly with age. In contrast, the most prominent signature in EOCRC was SBS87‐like, a COSMIC signature previously associated with thiopurine exposure. Because TCGA does not provide detailed thiopurine exposure, inflammatory bowel disease history, or treatment records, this finding should be interpreted as an association with an SBS87‐like mutational pattern rather than direct evidence of prior thiopurine treatment. It remains possible that the SBS87‐like signal reflects unmeasured environmental or dietary exposures, treatment in a small subset of patients, or instability of NMF decomposition in the limited EOCRC sample. Thus, the inferred signature composition should be considered exploratory rather than definitive.

Regarding the immune microenvironment, EOCRC exhibited a distinct immunosuppressive profile. CIBERSORT analysis showed significantly elevated Treg scores and increased resting NK cell fractions. CD8+ T‐cell fractions showed a borderline difference between groups (*p* = 0.0497), which we do not interpret as a clear absence of difference. Elevated Tregs are a well‐recognized mechanism of immune evasion. Furthermore, the positive correlation between immune scores and stromal scores was significantly stronger in EOCRC than in LOCRC, indicating tighter coupling between immune infiltration and desmoplastic stromal reaction. Spatial transcriptomics supported this by showing altered distribution patterns of COL1A1‐expressing fibroblasts alongside T‐cell, B‐cell, and NK/cytotoxicity markers in EOCRC tissues, suggesting that cancer‐associated fibroblasts may shape immune cell localization and function.

At single‐cell resolution, we dissected the myeloid compartment and identified subpopulations with divergent M1/M2 polarization ratios. Notably, SPP1 (osteopontin)‐expressing macrophages—which have been associated with tumor progression, angiogenesis, and immune suppression—were present in EOCRC [35, 36]. Myeloid‐derived suppression may therefore contribute to EOCRC biology, whereas macrophage‐ or SPP1‐directed strategies remain hypotheses requiring preclinical validation.

At the systems level, WGCNA identified a lightgreen module that showed the strongest positive correlation with EOCRC status. This module was significantly enriched in pathways related to mitochondrial gene expression, ribosome biogenesis, lipid and sterol biosynthesis, and protein localization to membranes. This transcriptional signature suggests that EOCRC tumors are metabolically reprogrammed toward anabolic activity and oxidative phosphorylation. The coenrichment of ribosome biogenesis and protein synthesis pathways is consistent with the aggressive clinical behavior often observed in EOCRC. The module also included terms such as methylation, Notch receptor processing, and positive regulation of telomere maintenance, which may link to epigenetic and telomeric alterations reported in early‐onset cancers. These findings identify potential drug targets, including inhibitors of mitochondrial translation, ribosome biogenesis, or lipid biosynthesis, pending preclinical validation.

Beyond the broad immunosuppressive phenotype described above, our analysis revealed substantial immune heterogeneity within EOCRC itself. Through hierarchical clustering of 29 immunogenomic signatures across the TCGA‐CRC cohort, we identified two distinct immune archetypes—cold‐ and hot‐like—among which the hot‐like subgroup was notably enriched for LOCRC, whereas most EOCRC tumors clustered into the cold‐like category. The FAT3‐mutant hot‐like subgroup therefore represents a minority within the generally immunosuppressive EOCRC landscape rather than a contradictory population‐level finding. Application of a random forest algorithm identified FAT3 as one of the top three most informative genes distinguishing hot‐like from cold‐like EOCRC, alongside APC and TRPS1. FAT3 mutations were enriched in hot‐like EOCRC (40.74%) relative to cold‐like EOCRC (3.57%), with alterations predominantly comprising missense and frameshift events. Moreover, FAT3‐mutant EOCRC exhibited elevated SNV neoantigens, nonsilent mutation rates, indel neoantigens, aneuploidy scores, and homologous recombination defects, alongside enhanced leukocyte fractions, IFN‐*γ* responses, and macrophage regulation. These findings suggest that FAT3 mutations may mark a distinct immune‐activated, hot‐like subgroup within EOCRC, potentially reflecting genomic instability and augmented neoantigen generation rather than a direct causal effect. FAT3 is a member of the FAT family of atypical cadherins and has been implicated in genomic stability and immune modulation in other malignancies. In gastric cancer, FAT3 mutations have been associated with prognosis and increased immune infiltration [37]. In lung adenocarcinoma, FAT3 alterations correlate with elevated TMB and favorable response to immune checkpoint inhibitors [38]. Endometrial cancer studies similarly report FAT3 mutations in the context of microsatellite instability and immune activation [39]. These cross‐tumor observations are consistent with our finding that FAT3‐mutated EOCRC exhibits genomic instability, enhanced neoantigen generation, and hot‐like immune features. Importantly, the single‐cell analysis further suggests that FAT3 expression is concentrated in CAFs, particularly myofibroblasts, rather than epithelial tumor cells. Thus, bulk‐tumor FAT3 mutation and stromal FAT3 expression represent distinct biological observations that should not be conflated. The convergence of these observations supports a testable role for FAT3‐related programs in modulating the TME, although the underlying mechanism remains incompletely understood. The observed association may also be partially confounded by the overall higher TMB in this subgroup, and multivariate validation in larger cohorts is required.

From a translational perspective, the findings can be prioritized into several testable levels. First, the association between FAT3 mutation and hot‐like immune features can be evaluated retrospectively in clinically annotated EOCRC cohorts with immune checkpoint inhibitor outcomes, ideally with multivariable adjustment for TMB, microsatellite instability, and other confounders. Second, the predicted FAT3‐dependent CAF programs require preclinical testing using CRISPR‐mediated FAT3 perturbation in myofibroblast CAFs and tumor‐CAF coculture or organoid models, with immune‐cell chemotaxis, extracellular matrix remodeling, and interferon‐response readouts. Third, the mitochondrial‐ribosomal biogenesis module and SPP1‐expressing myeloid programs remain exploratory therapeutic hypotheses that require pharmacologic perturbation and biomarker‐linked validation before clinical translation. Finally, the druggable‐target analysis is descriptive and should be used to prioritize candidates rather than to infer immediate clinical actionability.

Several limitations should be acknowledged. The sample size of EOCRC in the TCGA cohort (*n* = 69) is not large, although key mutation findings were validated in an independent DFCI cohort. The association between the SBS87‐like signature and prior thiopurine therapy requires confirmation with detailed clinical histories, including treatment exposure and inflammatory bowel disease status. Spatial transcriptomics data were derived from a limited number of tissue sections and may not capture full intratumoral heterogeneity. The CD8+ T‐cell comparison was borderline (*p* = 0.0497) and should be interpreted cautiously. The in silico FAT3 perturbation analysis is computational and hypothesis generating; it does not constitute functional validation, and FAT3 mutation status in bulk tumors is not equivalent to FAT3 expression or loss of function in myofibroblast CAFs. Experimental perturbation in CAF, organoid, or coculture systems and validation in clinically annotated EOCRC cohorts are needed to establish causality. Finally, the upstream regulators of the lightgreen module remain unknown.

In summary, our multiomic analysis reveals that EOCRC represents a molecularly heterogeneous entity distinct from LOCRC, characterized by different mutational‐signature patterns—notably an SBS87‐like signal in EOCRC versus age‐related clock‐like deamination in LOCRC—and by an immunosuppressive, stroma‐coupled microenvironment with M2‐polarized myeloid infiltration. At the systems level, EOCRC exhibits a unique transcriptional program centered on mitochondrial and ribosomal biogenesis, suggesting metabolic reprogramming toward anabolic activity. Importantly, within the generally immune‐depleted landscape of EOCRC, we identify FAT3 mutations as a candidate genomic correlate of a hot‐like, immune‐activated subgroup associated with genomic instability and enhanced neoantigen generation. Integrative single‐cell and in silico perturbation analyses further nominate FAT3‐related myofibroblast CAF programs as testable mechanistic hypotheses. Collectively, these findings provide a molecular framework for understanding early‐onset disease and prioritize biomarker and therapeutic hypotheses that now require functional validation and testing in clinically annotated cohorts.

## 5. Conclusions

EOCRC is molecularly heterogeneous and distinct from LOCRC, with an SBS87‐like mutational pattern, an immunosuppressive microenvironment with Treg enrichment, and tight immune‐stromal coupling. Our findings nominate FAT3 mutations as a potential genomic correlate of a hot‐like, immune‐activated EOCRC subgroup characterized by genomic instability and enhanced neoantigen generation, whereas integrative single‐cell and in silico perturbation analyses nominate FAT3‐related myofibroblast CAF programs as additional hypotheses. These observations are hypothesis generating and require functional validation in experimental models and independent clinical cohorts. Together, they provide a framework for future age‐stratified precision‐oncology studies in younger patients.

## Author Contributions

Conceptualization: Liping Wang and Yanjun Lu. Data curation: Liping Wang, Yanjun Lu, and Weiyu Ma. Formal analysis: Liping Wang, Yanjun Lu, Jinfang Guo, and Weiyu Ma. Supervision: Wenbin Zhang and Yinan Shi. Writing—original draft: Liping Wang and Yanjun Lu. Writing—review and editing: Wenbin Zhang and Yinan Shi. Liping Wang and Yanjun Lu contributed equally to this work and should be listed as co‐first authors.

## Funding

No funding was received for this manuscript.

## Disclosure

All authors have read and approved the final manuscript.

## Ethics Statement

This study relied exclusively on published or public datasets; therefore, no ethical approval or consent was necessary.

## Conflicts of Interest

The authors declare no conflicts of interest.

## Data Availability

The data that support the findings of this study are available from the corresponding authors upon reasonable request.
